# Evaluating the Effectiveness of Implementing a More Severe Drunk-Driving Law in China: Findings from Two Open Access Data Sources

**DOI:** 10.3390/ijerph14080832

**Published:** 2017-07-25

**Authors:** Wangxin Xiao, Peishan Ning, David C. Schwebel, Guoqing Hu

**Affiliations:** 1 Department of Epidemiology and Health Statistics, Xiangya School of Public Health, Central South University, Changsha 410078, China; xiaowangxin@csu.edu.cn (W.X.); ningpeishan@csu.edu.cn (P.N.); 2 Department of Psychology, University of Alabama at Birmingham, Birmingham, AL 35294, USA; schwebel@uab.edu

**Keywords:** drunk-driving, law, mortality, morbidity, Global Burden of Disease (GBD), police data, alcohol use

## Abstract

In 2011, China implemented a more severe drunk-driving law. This study evaluated the effectiveness of the law on road traffic morbidity and mortality attributed to alcohol use. Data were from two open-access data sources, the Global Burden of Disease (GBD) 2015 update and police data. Poisson regression examined the significance of changes in morbidity and mortality. Large gaps in crude death estimates from road traffic crashes attributed to alcohol use emerged between the two data sources. For the GBD 2015 update, crude and age-standardized mortality displayed consistent trends between 1990 and 2015; age-standardized mortality per 100,000 persons increased from 5.71 in 1990 to 7.48 in 2005 and then continuously decreased down to 5.94 in 2015. Police data showed a decrease for crude mortality per 100,000 persons from 0.29 in 2006 to 0.15 in 2010 and then an increase to 0.19 in 2015. We conclude available data are inadequate to determine the effectiveness of the implementation of the more severe drunk-driving law in China since the two data sources present highly inconsistent results. Further effort is needed to tackle data inconsistencies and obtain reliable and accurate data on road traffic injury attributable to alcohol use in China.

## 1. Introduction

Alcohol use is a major risk factor for road traffic crashes worldwide. According to the Global Burden of Disease (GBD) 2015 update, 0.33 million road traffic deaths can be attributed to alcohol use globally in 2015 [1]. In China, about 30% of road traffic deaths, or 93,750 fatalities, were ascribed to drunk-driving in 2015 [1].

As a cost-effective intervention [2], implementation of drunk-driving law was included by the United Nations in the list of recommended interventions for member countries in *The Global Plan for the Decade of Action for Road Safety 2011–2020* [3,4]. In China, the Standing Committee of the National People’s Congress amended the Road Traffic Safety Law of the People’s Republic of China in 2011 to enhance the penalties for drunk-driving [5,6]. At that point, drunk driving became a criminal offence, with severe punishments issued depending on the severity of the crime [7,8]. Specifically, when drivers are detected having a blood alcohol concentration (BAC) of 0.02 to 0.08 g/100 mL, they are charged with drink driving and their license will be suspended for 5 years. When a driver is detected having a BAC > 0.08 g/100 mL, drunk driving is charged. Drunk driving is deemed as a crime in China and may cause imprisonment and suspension of a driving license for 10 years. In the case of a fatal traffic crash and a drunk driving charge, driving licenses may be suspended for life [5,8].

Timely and rigorous evaluative studies are critical to monitor the effectiveness of implementation of drunk-driving law, but few publications are available [9]. No published studies in English or Chinese examine the effectiveness of the implementation of drunk-driving law in China. Thus, we sought to accomplish this goal by examining data from two open access data sources, the GBD 2015 update and Chinese police data, that provide estimates of road traffic morbidity and mortality attributable to alcohol use for China. Multiple data sources offer stronger evidence than a single data source if they agree with each other. In this present study, therefore, we used both data sources to assess the effectiveness of drunk-driving law implementation in China. We hypothesized there might be sharp decreases in alcohol-related traffic injuries following implementation of the more severe drunk-driving law in China in 2011.

## 2. Materials and Methods

### 2.1. Ethical Statement

This study was completely based on public access data, and therefore it was exempted from ethics review. The research plan was approved by the Medical Ethics Committee of Central South University (No. XYGW-2017-25).

### 2.2. Data Sources

The GBD 2015 update data were accessed through the online visualization tool “GBD Compare, Viz Hub” [1], which provides estimates of crude and age-standardized morbidity and mortality rates for road traffic injuries attributable to alcohol use at six annual time points (1990, 1995, 2000, 2005, 2010 and 2015). The “GBD Compare” is an interactive online data visualization tool created by the Institute for Health Metrics and Evaluation at the University of Washington, providing updated data about the estimates of major health outcome indicators at global, national and subnational levels from 1990 to 2015. The GBD 2015 study group employs various analytical tools and a diverse set of data sources to generate comparable estimates and 95% uncertainty intervals of deaths and mortality rates broken down by age, sex, cause, year, and geography [10]. The GBD 2015 update uses the 10th International Classification of Diseases (ICD-10) to record diseases and injuries. ICD-10 codes for road traffic injury include V01-V04.99, V06-V80.929, V82-V82.9, V87.2-V87.3 [10].

Police data are from the Statistical Yearbook of Road Traffic Accidents in China [11]. Police data were collected by road traffic police officers using a standardized data collection questionnaire that consists of the number of crude road traffic deaths and injuries from drunk driving. Despite the argument that police data may seriously undercount the number of road traffic deaths and injuries [11,12,13], they are the only data source that collects direct evidence on the number of drunk driving incidents in China. Therefore, the data have value in assessing trends in morbidity and mortality. 

Population data for calculating crude morbidity and mortality rates were derived from the China Statistical Yearbook 2016 [14].

### 2.3. Data Analysis

Because both data sources present morbidity and mortality rates, we compared changes in rates before and after the implementation of the more severe drunk-driving law in 2011 to assess effectiveness of the law. We selected 2010, the year before the more severe drunk-driving law was implemented, as the reference year to calculate the relative risk (RR) of morbidity and mortality in the years before and after 2010. The GBD 2015 update provides a surrogate morbidity indicator, years lived with disabilities (YLDs) per 100,000 persons. YLDs are estimated by the product of the number of prevalent cases with a certain health outcome and the disability weight for the health outcome. Disability weights, ranging from 0 (equivalent to full health) to 1 (equivalent to death), are estimated through expert judgment [15].

We graphed crude and age-standardized morbidity (YLDs per 100,000 persons) and mortality from 1990 to 2015 for the GBD 2015 update and crude morbidity (incidence per 100,000 persons) and mortality from 2006 to 2015 for the police data. 95% uncertainty interval (95% UI) was also included for GBD 2015 estimates. Poisson regression was used to examine the significance of changes in morbidity and mortality. Data analysis was performed using Microsoft Excel 2010 (Microsoft, Albuquerque, NM, USA) and Stata/IC 12.1 (Stata Corp LLC, College Station, TX, USA).

## 3. Results

### 3.1. Differences in Crude Deaths

Both the GBD 2015 update and the police data provide alcohol-related crude road traffic deaths from three years: 2005, 2010, and 2015. The crude deaths estimates from the GBD 2015 update ranged from 23–53 times those of police data (Table 1). 

### 3.2. GBD 2015 Update

**Figure 1:**
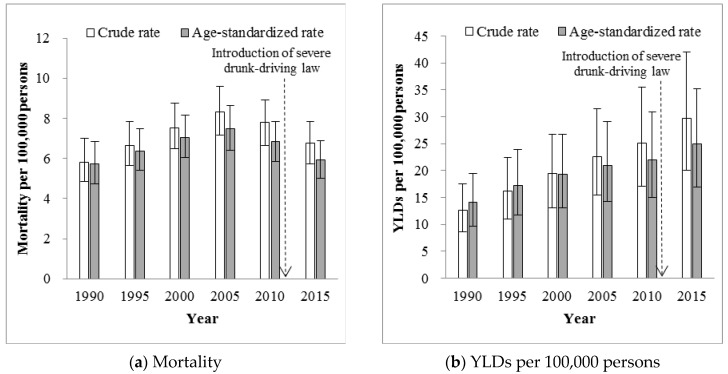
Crude and age-standardized mortality and years lived with disabilities (YLDs) per 100,000 persons from road traffic crashes attributed to alcohol use in China based on Global Burden of Disease (GBD) 2015 update, 1990–2015. China implemented more severe penalties for drunk driving in 2011. Error bars denote 95% uncertainty intervals of the estimates.

According to the GBD 2015 update, both crude and age-standardized mortality follow a consistent pattern from 1990 to 2015 (Figure 1a), with age-standardized mortality attributed to alcohol use first increasing gradually between 1990 and 2010 and then decreasing between 2010 and 2015. Compared to 2010, the year before the more severe penalty for drunk driving was implemented, the RR for 1990, 1995, 2000, 2005 and 2015, respectively, was 0.8319, 0.9275, 1.0267, 1.0897 and 0.8651, all *p* < 0.05 (Table 2). 

Crude and age-standardized morbidity (YLDs per 100,000 persons) also presented consistent patterns from 1990 to 2015 (Figure 1b). In contrast to the pattern for age-standardized mortality, age-standardized morbidity showed a continuously increasing trend across the full study time period. Using 2010 as reference year, the RR of morbidity from drunk driving increased from 0.6428 in 1990 to 1.1328 in 2015, all *p* < 0.05 (Table 2). Crude data followed similar patterns.

### 3.3. Police Data

**Figure 2:**
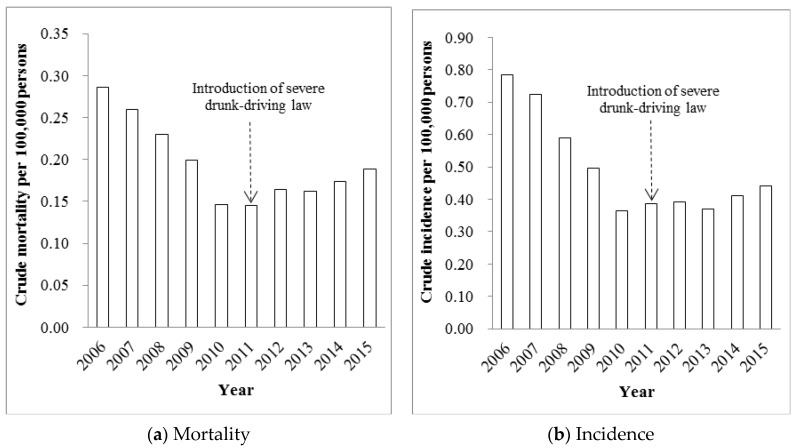
Crude mortality and incidence per 100,000 persons from road traffic crashes attributed to alcohol use in China based on police data, 2006–2015. Published police data only covered crude numbers of deaths and injuries from 2006 to 2015 from road traffic crashes attributed to alcohol use. China implemented the more severe drunk driving law in 2011.

Police data showed a somewhat different pattern compared to the GBD 2015 Update data. Using crude mortality and incidence for police data, we observed highly consistent changes over time from 2006 to 2015 (Figure 2a,b). Both crude mortality and incidence from drunk driving decreased from 2006 to 2010, and then presented a gradual increasing tendency with small fluctuations from 2011 to 2013.

Compared to 2010, the RR of crude mortality first decreased from 1.9376 in 2006 to 1.3624 in 2009, and then increased from 1.0002 in 2011 to 1.3397 in 2015 (Table 3). Similarly, the RR of incidence first dropped from 2.1014 in 2006 to 1.3444 in 2009 and then rose from 1.0614 to 1.2419 during 2011–2015.

## 4. Discussion

Our study offers three major findings, all of them intriguing but somewhat challenging to interpret. First, the GBD 2015 update and police data revealed significant disparities in the number of road traffic injuries attributed to alcohol use, as well as inconsistent patterns in rates of morbidity and mortality attributable to alcohol use after the implementation of more severe national drunk-driving law in 2011. Second, the GBD 2015 update displayed inconsistent changes in age-standardized morbidity versus mortality rates before and after the implementation of the more severe national drunk-driving law in 2011, with morbidity changing in the positive direction (13.28%) between 2010 and 2015 but mortality changing in the negative direction (−13.49%). Third, police data suggest a steady decrease in both crude morbidity and mortality before the implementation of a more severe drunk-driving law (from 2006 to 2010), but a gradual increase after 2011. We discuss each of these findings below.

The inconsistent findings between GBD 2015 update and police data are probably a result of the different strategies each dataset used to estimate injuries attributable to alcohol use. The GBD 2015 update applies sophisticated mathematical modeling based on various available data sources to generate estimates [10], while police data are collected through a standardized questionnaire after each crash related to drunk driving is suspected by a police officer. Each data source offers both advantages and disadvantages, and it seems likely that neither is perfectly valid. The GBD 2015 update provides estimates based on risk factor attribution models and available research evidence. Because there is a lack of high-quality, continuous exposure data documenting fatal and non-fatal injury data in China [15,16], the death estimates attributable to alcohol use in the GBD 2015 update may be biased to some extent. The police data consist of the actual numbers of fatal and non-fatal injuries attributable to crashes from drunk driving that are directly collected by road police. Police data are documented to have serious under-reporting problems for a variety of reasons [12,17].

The fact that morbidity and mortality data showed inconsistent changes in the GBD 2015 update may primarily be a result of data input strategies for the estimation models. In the GBD 2015 update, the major input data to estimate morbidity in China are surveillance data from the National Injury Surveillance System (NISS). Because the NISS was established only in 2006 [16], the GBD 2015 update employs a combination of advanced models and fragmented published data to estimate numbers and rates of non-fatal injury in China. In addition, NISS is a hospital-based surveillance system and its surveillance data are highly sensitive to health policy changes that lead to substantial changes in people seeking hospital-based medical care. For example, as the governmental investment increased, the coverage of social medical health insurance increased from 85% in 2008 to 95% in 2010, and 97% in 2015 [18,19,20]. The rise of social medical insurance coverage led to a jump of patients who visited public hospitals and resulted in an impact on injury surveillance data [21,22].

The result that police data indicate increasing drunk driving incidents following implementation of the more severe penalties but decreasing rates beforehand is bewildering. It may be caused by a combination of at least four factors: (a) rapid motorization and an increase in licensed motor vehicle drivers that occurred across China during that era, leading to more vehicles on the road and greater opportunity for alcohol-related road traffic crashes; (b) public education and enforcement that accompanied implementation of the more severe drunk-driving law, perhaps increasing recognition and recording of incidents; (c) other prevention efforts, including improvements of the road environment and introduction of new safety devices in motor vehicles, and/or (d) changes in data reporting that occurred, partly because of increased public education about and attention toward alcohol-related road traffic crashes nationwide during that time period. Data on enforcement seem particularly intriguing as an explanation. After implementing the severe drunk driving law, the traffic police division within the Ministry of Public Security of China greatly increased enforcement of drunk driving law [23]. For example, the number of roadside breathing tests for drunk driving increased from 0.46 million to 0.78 million and the number of drivers whose licenses were temporarily suspended due to drunk driving grew from 0.33 million to 0.50 million from 2011–2015 [11].

We conclude that the currently available data are insufficient to judge the effectiveness of implementing the more severe drunk driving law in China. Both the GBD 2015 update and police data suffer from some flaws; neither provides strong evidence of our hypothesized effect. The results highlight the importance and urgency of establishing a high-quality free and online data query system to support policy evaluation in China. China could replicate the successful establishment of the Fatality Analysis Reporting System (FARS) in the United States, which was created in 1975 and is now widely used to enact and evaluate road traffic laws including child restraint laws, state safety belt laws and Graduated Driving Licensing (GDL) laws [24]. The data and results do, however, indicate the possibility of behavior change occurring as a result of the law. Enforcement may have increased, and publicity and government effort to detect dangerous driving are documented to have increased [11,23,25,26]. Data from other nations offer clear evidence that reducing drunk-driving rates requires a multi-faceted effort, including safety education, institution of more severe penalties for risky driving under the influence of alcohol, and strong enforcement of law.

## 5. Limitations

This study is primarily limited by the quality and availability of data. Because the data lack all relevant explanatory variables, we cannot definitively interpret the reasons for the inconsistent findings. Further efforts to improve data quality are needed.

## 6. Conclusions

Data from two available sources—the GBD 2015 update and national police data—display large inconsistencies in the morbidity and mortality of road traffic injuries attributed to alcohol use in China. Both data sources offer results that are difficult to interpret, and the results across data sources are inconsistent with each other. We conclude there is currently weak evidence to indicate implementation of the more severe drunk-driving law in China resulted in a decrease in road traffic morbidity and mortality attributable to alcohol use. We offer this conclusion with the caveat that the current data sources are inadequate to evaluate the effectiveness of drunk-driving law in China, however, and encourage further research to explore the reasons behind the inconsistency between the two data sources and efforts to improve data quality on road traffic injury, including injury attributable to alcohol use, in China.

## Figures and Tables

**Table 1:** Crude deaths from road traffic crashes attributed to alcohol use in China.

Year	GBD 2015 Update (A)	Police Data (B)	Ratio (A/B)
2005	109,238	4715	23
2010	105,396	1998	53
2015	93,750	2744	34

Note: Number of crude deaths from police data only included those from motor vehicle crashes attributed to alcohol use in 2005; GBD: Global Burden of Disease.

**Table 2:** Changes in crude and age-standardized mortality and YLDs per 100,000 persons from road traffic crashes attributed to alcohol use in China based on GBD 2015 update, 1990–2015.

Type of Data	Year	Mortality		YLDs per 100,000 Persons	
		RR	95% CI	RR	95% CI
Crude data	1990	0.7454 *	(0.7429, 0.7479)	0.5029 *	(0.5018, 0.5040)
	1995	0.8525 *	(0.8498, 0.8552)	0.6461 *	(0.6449, 0.6473)
	2000	0.9649 *	(0.9620, 0.9678)	0.7712 *	(0.7698, 0.7726)
	2005	1.0650 *	(1.0619, 1.0681)	0.9012 *	(0.8996, 0.9027)
	2010	1.0000		1.0000	
	2015	0.8674 *	(0.8648, 0.8700)	1.1798 *	(1.1780, 1.1817)
Age-standardized data	1990	0.8319 *	(0.8291, 0.8346)	0.6429 *	(0.6415, 0.6442)
	1995	0.9275 *	(0.9246, 0.9304)	0.7865 *	(0.7850, 0.7880)
	2000	1.0267 *	(1.0236, 1.0298)	0.8760 *	(0.8744, 0.8775)
	2005	1.0897 *	(1.0865, 1.0928)	0.9527 *	(0.9511, 0.9543)
	2010	1.0000		1.0000	
	2015	0.8651 *	(0.8624, 0.8677)	1.1328 *	(1.1310, 1.1345)

* *p* < 0.05; RR: Relative risk; YLDs:years lived with disabilities.

**Table 3:** Changes in crude mortality and incidence from road traffic crashes attributed to alcohol use in China based on police data, 2006–2015.

Year	Mortality		Incidence	
	RR	95% CI	RR	95% CI
2006	1.9376 *	(1.9018, 1.9741)	2.1014 *	(2.0773, 2.1258)
2007	1.7585 *	(1.7255, 1.7921)	1.9410 *	(1.9184, 1.9638)
2008	1.5691 *	(1.5391, 1.5998)	1.5899 *	(1.5707, 1.6092)
2009	1.3624 *	(1.3355, 1.3897)	1.3444 *	(1.3277, 1.3614)
2010	1.0000		1.0000	
2011	1.0002	(0.9791, 1.0217)	1.0614 *	(1.0475, 1.0755)
2012	1.1316 *	(1.1084, 1.1552)	1.0753 *	(1.0613, 1.0895)
2013	1.1285 *	(1.1054, 1.1520)	1.0311 *	(1.0176, 1.0449)
2014	1.2188 *	(1.1943, 1.2437)	1.1513 *	(1.1366, 1.1663)
2015	1.3397 *	(1.3134, 1.3665)	1.2419 *	(1.2262, 1.2577)

RR: Relative risk, * *p* < 0.05.
